# Multiple Probe Measurements at Uranus Motivated by Spatial Variability

**DOI:** 10.1007/s11214-024-01050-9

**Published:** 2024-02-09

**Authors:** Michael H. Wong, Naomi Rowe-Gurney, Stephen Markham, Kunio M. Sayanagi

**Affiliations:** 1 Center for Integrative Planetary Science, University of California, Berkeley, CA 94720-3411 USA; 2 Carl Sagan Center for Science, SETI Institute, Mountain View, CA 94043-5232 USA; 3 NASA Goddard Space Flight Center, Greenbelt, MD 20771 USA; 4 University of Maryland, College Park, MD 20742 USA; 5 The Center for Research and Exploration in Space Science & Technology (CRESST II), Greenbelt, MD 20771 USA; 6 The Royal Astronomical Society, Piccadilly, London, W1J 0BD UK; 7 Observatoire de la Côte d’Azur, 06300 Nice, France; 8 Department of Astronomy, New Mexico State University, Las Cruces, NM 88003 USA; 9 NASA Langley Research Center, Hampton, VA 23681 USA

**Keywords:** Uranus, Atmospheric probes, Planetary atmospheres, Spatial variability, Giant planets, Planet formation

## Abstract

A major motivation for multiple atmospheric probe measurements at Uranus is the understanding of dynamic processes that create and maintain spatial variation in thermal structure, composition, and horizontal winds. But origin questions—regarding the planet’s formation and evolution, and conditions in the protoplanetary disk—are also major science drivers for multiprobe exploration. Spatial variation in thermal structure reveals how the atmosphere transports heat from the interior, and measuring compositional variability in the atmosphere is key to ultimately gaining an understanding of the bulk abundances of several heavy elements. We review the current knowledge of spatial variability in Uranus’ atmosphere, and we outline how multiple probe exploration would advance our understanding of this variability. The other giant planets are discussed, both to connect multiprobe exploration of those atmospheres to open questions at Uranus, and to demonstrate how multiprobe exploration of Uranus itself is motivated by lessons learned about the spatial variation at Jupiter, Saturn, and Neptune. We outline the measurements of highest value from miniature secondary probes (which would complement more detailed investigation by a larger flagship probe), and present the path toward overcoming current challenges and uncertainties in areas including mission design, cost, trajectory, instrument maturity, power, and timeline.

## Introduction

The Galileo Probe was the first and only atmospheric entry probe to explore a giant planet atmosphere (Young 2003). Surprises in the vertical profiles of temperature and volatile gases retrieved by the probe led researchers to call for multiple entry probes on future missions (Owen et al. 1997; Atreya et al. 1999; Atreya and Wong 2005; Atkinson et al. 2009). Challenges still remain to this day when trying to interpret Galileo profiles in the context of spatial variability retrieved from more recent remote sensing of Jupiter (Sect. 4). In response to the Galileo Probe discoveries, the first planetary science decadal survey (National Research Council 2003, hereafter *New Frontiers* 2003) recommended that future probe missions to Jupiter, Uranus, and Neptune include multiple probes. Multiprobes were part of the second New Frontiers Announcement of Opportunity (NF2 AO), released at the end of 2003^1^ by the National Aeronautics and Space Administration (NASA). The NF2 AO included a mission category for “Jupiter Polar Orbiter with Probes.”

By the time of publication of the second planetary decadal survey (National Research Council 2011, hereafter *Visions and Voyages* 2011), the Juno mission (Bolton et al. 2017) had been launched, with a plan to achieve the preponderance of Jupiter Polar Orbiter with Probes science goals using an orbiter alone. Compared to New Frontiers 2003, Visions and Voyages 2011 considered cost more thoroughly, and was more reserved in its endorsement of multiprobes. It discussed a New Frontiers class Saturn Probe mission, considering multiprobes “to further enhance the science yield” but not including them in the baseline mission concept study. A Uranus Orbiter and Probe (UOP) mission was recommended to start in the 2013–2022 decade, but with lower priority than Mars Astrobiology Explorer-Cacher and Jupiter Europa Orbiter (Visions and Voyages 2011).

The most recent decadal survey completely avoided all mention of multiprobes to the giant planets (National Academies of Sciences, Engineering, and Medicine 2022, hereafter *Origins, Worlds, and Life* 2022). This survey recommended a UOP mission as the next high priority Flagship mission for NASA.

Strong science drivers remain for multiple atmospheric probes to the giant planets (particularly Uranus, as discussed by Fletcher et al. 2020), despite the changing level of explicit support from survey to survey over the past three decades. In this paper, we present the overarching science drivers for including multiple probes on the UOP mission (Sect. 2). We support these drivers with a detailed review of spatial variability in the atmosphere of Uranus, covering the current state of knowledge and open questions (Sect. 3). In Sect. 4 we discuss considerations at the other giant planets which continue to justify multiprobe exploration there and which provide examples of the more complete science at Uranus that could be achieved using multiple probes. We list the impactful but technically modest set of measurements desired from secondary probes (Sect. 5), and provide potential solutions to challenges that are of concern for multiprobe missions (Sect. 6).

## Science Drivers for Multiprobes

The decadal survey described a research strategy to advance the frontiers of planetary science based on several Priority Science Questions, each broken up into multiple sub-questions (Origins, Worlds, and Life 2022). The obvious question for atmospheric probe investigations is Q7: Giant Planet Structure and Evolution, but probe measurements of heavy elements provide important constraints for origin questions Q1: Evolution of the Protoplanetary Disk, and Q2: Accretion in the Outer Solar System. Table 1 lists the decadal survey science questions that are addressed by multiprobe investigations of Uranus.

**Table 1:** Priority Science Questions from Origins, Worlds, and Life 2022

Number	Question
*Q1*	*Evolution of the Protoplanetary Disk*
Q1.1	What were the initial conditions in the solar system?
Q1.1c	How did the compositions of the gas, dust, ice and organic components, and the physical conditions vary across the protoplanetary disk?
Q1.2	How did distinct reservoirs of gas and solids form and evolve in the protoplanetary disk?
Q1.3	What processes led to the production of planetary building blocks i.e., planetesimals?
Q1.4	How and when did the nebula disperse?
Q1.4b	What mechanisms dispersed the nebula?
*Q2*	*Accretion in the Outer Solar System*
Q2.1	How did the giant planets form?
Q2.2	What controlled the compositions of the material that formed the giant planets?
Q2.2c	How were compositional differences between the gas giants and ice giants influenced by the chemical and physical processing of accreted solids and gas?
*Q7*	*Giant Planet Structure and Evolution*
Q7.1	What are giant planets made of and how can this be inferred from their observable properties?
Q7.2	What determines the structure and dynamics deep inside giant planets and how does it affect their evolution?
Q7.3	What governs the diversity of giant planet climates, circulation, and meteorology?
Q7.5	How are giant planets influenced by, and how do they interact with, their environment?
Q7.5b	How is atmospheric composition influenced by ring rain, large impacts, and micrometeoroids?^a^

^a^Science question overlaps with Q4.3e: What exogenic volatile and non-volatile materials are delivered to planetary bodies?

All of the questions in Table 1 would be addressed by a single atmospheric probe (Dahl et al. 2024; Mandt et al. 2024); the fact that secondary probes also address these questions does not imply that they can *only* be addressed by multiple probes. But completely solving any of the Priority Science Questions is a very long-term goal, ultimately requiring in-situ sampling of the atmospheres of all four giant planets, as well as atmospheric remote sensing utilizing spectroscopy, imaging, and time-series data across the spectrum (Simon et al. 2022; Roman 2023), observations of exoplanets and protoplanetary disks, characterization of solar system small bodies and their populations, and ongoing studies of satellites and ring systems. The motivation for multiprobe exploration comes from the range of unique advances over exploration using a single probe.

### Origins

For some compositional measurements central to questions of planetary origins—particularly noble gas abundances and isotope ratios—atmospheric concentrations are not thought to vary spatially, so there is no advantage provided by a second probe (Mandt et al. 2024). But volatile elements C, O, N, and S are valuable tracers of planet formation, and they are found in atmospheric molecules with spatially varying concentrations. Secondary probes thus have the important role of quantifying spatial variability so as to ultimately establish the most representative values of atmospheric composition as a tracer of planet formation.

**Fig. 1:**
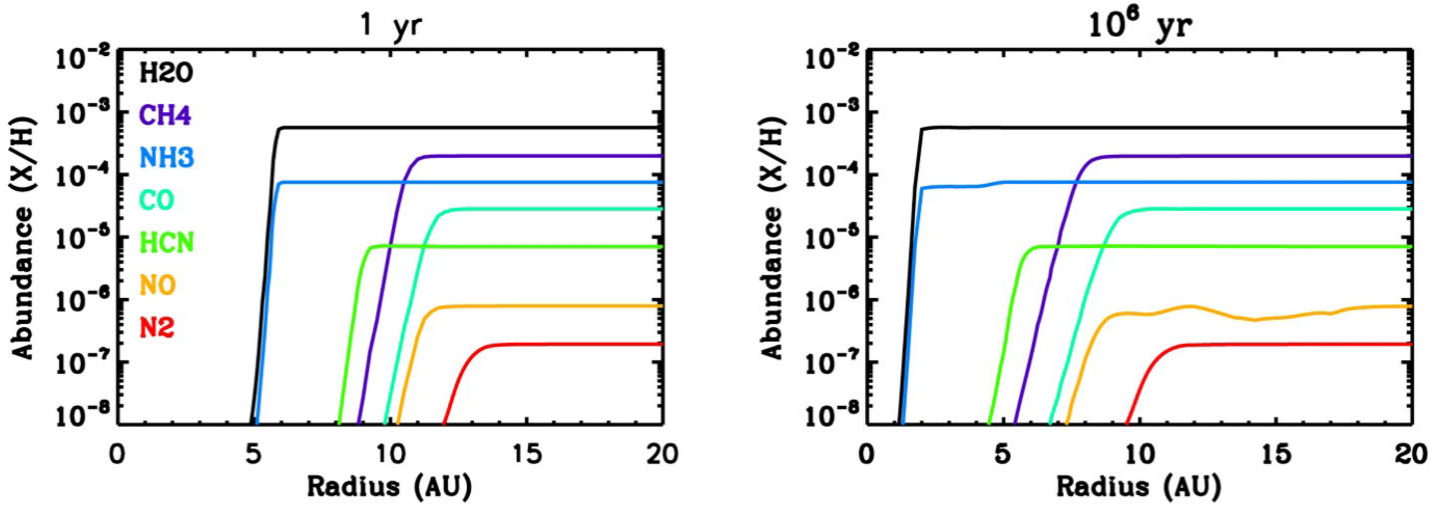
Ice abundances as a function of radial distance in the model of Dodson-Robinson et al. (2009), at the start of the calculation and after a million years. Ice lines for different molecular species moved inwards as the disk cooled, affecting the inventory of solid materials available to form planetesimals and pebbles ultimately accreted by the giant planets as they formed

The bulk composition of Uranus tracks the complex and dynamic conditions in the protoplanetary disk. Spatially, composition as a function of radial distance from the Sun evolved over time (Fig. 1), as controlled by snow lines and condensation fronts of different volatile species. The partitioning between components such as gas, dust, ice, and organics varied spatially, and these components had distinct processes of transport, loss, and production. Ultimately, any model of planet formation within the inhomogeneous protoplanetary disk must be consistent with the current composition of Uranus. The decadal survey Strategic Research for understanding spatially variable conditions across the disk (Q1.1) called out the importance of “in situ ... measurements of the elemental and isotopic composition of... atmospheres of bodies formed from different nebular reservoirs (especially Uranus).”

A wide range of processes operating within the protoplanetary disk affected the formation and evolution of gas and solid reservoirs (Q1.2, Q1.3, Q1.4). Outward migration of Uranus may have allowed it to reach its current mass before the dispersal of the protoplanetary disk, as in the model of Dodson-Robinson and Bodenheimer (2010), which achieves consistency with estimates of Uranus’ carbon mass fraction by carefully considering the planet’s accretion and migration history with respect to the methane ice line (Fig. 1). For gas reservoirs, processes such as sublimation and condensation would have set elemental ratios with respect to snowline locations, which evolved over time (Öberg et al. 2011; Mandt et al. 2020; Öberg and Bergin 2021). These elemental ratios then would have been preserved in Uranus and other modern solar system bodies. Elemental and isotopic ratios would have tracked the evolution and eventual dispersion of the disk due to radiative processing and escape, or photoevaporation (Guillot and Hueso 2006). For solids, the differing trapping efficiencies in amorphous and crystalline water ices (which are stable at colder/warmer temperatures, respectively) may affect the composition of pebbles and planetesimals accreted into the planets, through the relative abundances of oxygen and other volatiles (Bar-Nun et al. 1987; Hersant et al. 2004; Mousis et al. 2018), and some protostellar ice components could have even remained pristine within large (100 $\mu $m) grains (Bergner and Ciesla 2021). Strategic Research in the decadal survey includes measurements “especially for the ice giants” focusing on “elemental and stable isotopic compositions of refractory and volatile elements.” Here, comparing the composition of all four giant planets is key, since it seems that Jupiter and Saturn easily crossed the threshold for runaway gas accretion, while Uranus and Neptune may have approached it only as the nebula dispersed (Helled 2023). This drives the Strategic Research focused on “in situ measurement of the volatile elemental compositions” of the planets.

**Fig. 2:**
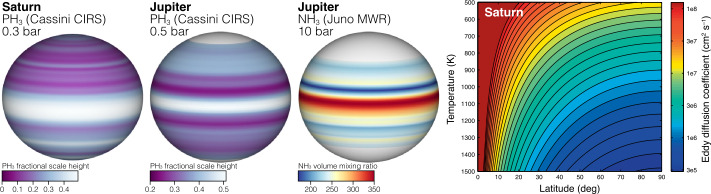
The Jupiter and Saturn cases demonstrate the need for new observations of the deep spatial variation of disequilibrium species, which can be used to constrain the bulk atmospheric abundance of oxygen. Left: Both Saturn and Jupiter have strong latitudinal banding in their PH_3_ distributions (Fletcher et al. 2009). For Jupiter there is a qualitative resemblance between the PH_3_ distribution at $P <$ 1 bar and the NH_3_ distribution at 10 bar (from Li et al. 2017). Right: Wang et al. (2015) found that deep eddy mixing was spatially variable due to planetary rotation, but the pattern of variability is less complex than the observations of PH_3_ at shallow levels

The specific needs for probe compositional measurements at multiple locations should be clear. The planetary C/O ratio provides an example (Cavalié et al. 2020, 2024), since the carbon abundance is measured from atmospheric CH_4_, which is known to vary spatially (Karkoschka and Tomasko 2009; Sromovsky et al. 2019a; James et al. 2022). Although methane has been measured from remote sensing, the range of atmospheric abundances from different analyses is large (Karkoschka and Tomasko 2009; Sromovsky et al. 2011, 2019b; Atreya et al. 2020), so in situ measurements in two locations would help to break remote sensing degeneracies affecting both the retrieved abundances as well as the spatial variability (Sect. 3). Atmospheric entry probes are unlikely to reach depths where oxygen (primarily in H_2_O) can be directly measured, but constraints can be placed by measurement of CO, a carrier of oxygen that is in thermochemical equilibrium only at much deeper levels. Mixing from these deep levels must be understood in order to use CO as a marker of the oxygen abundance, but again, spatially variable mixing in a global sense (Wang et al. 2015) will be easier to model with compositional measurements at different locations. Spatially-resolved in-situ measurements of PH_3_—which has not been detected in the troposphere from remote sensing, in part because it may condense near 1 bar (Encrenaz et al. 1996, 2004)—would help to break degeneracies between deep transport and deep abundance that must be understood to interpret CO data (Fig. 2).

Aside from questions about conditions across the protoplanetary disk over time, compositional measurements at Uranus also help us to understand the processes by which the giant planets accreted the disk material during their formation (Q2.1, Q2.2). Because there is no class of currently known solid material, whether icy or rocky, that follows the generally 3 times supersolar enrichment of heavy elements at Jupiter (Owen and Encrenaz 2003), it may be possible that focusing on understanding protoplanetary disk material alone may not answer the origins question. Materials accreted into the giant planets may have also been processed, through interior processes such as differentiation, mixing, and chemistry. The location of the planets may have determined the mix of materials that was accreted, since dynamical properties of the trans-Neptunian belt suggest that Neptune and Uranus migrated outward from a formation location closer to the Sun. Strategic Research for planetary accretion process questions again called for “in situ sampling of noble gas, elemental, and isotopic abundances.” Of particular importance for multiple probe measurements is the Strategic Research objective to “understand how compositional gradients in the atmosphere and interior of Jupiter, Saturn, Uranus, and Neptune affect the determination of bulk planetary composition based on observed atmospheric composition.” Atmospheric structure measurements were also considered strategic for this question, since the relevant data—“physical properties and boundary conditions (i.e., tropospheric temperatures, shapes, rotation rates) for structure models of Uranus and Neptune via... atmospheric profile measurements”—are important for understanding the deep structure and mixing in Uranus.

### Dynamic Processes

For many years, planetary scientists assumed that condensing vapor in convective fluid planets should be well-mixed below the cloud-forming level, and that the temperature structure below optical depth of order unity should be adiabatic. However, our experiences on Jupiter have challenged the validity of this “well-mixed assumption.” The atmosphere plays a fundamental role in a giant planet’s thermal evolution, because primordial heat must be transported by/through the atmosphere as it escapes to space. Dynamic processes engender spatial variation, making this science theme the obvious target for multiple probes.

Understanding the mapping between observable atmospheric properties and bulk planetary composition is central to both dynamical processes (Q7.1), as well as the origins topics discussed above. Compositional variation (horizontal and vertical) results from a balance between chemical processes (thermochemistry in the deep troposphere, cloud chemistry in the upper troposphere, and photochemistry in the stratosphere) and dynamical transport (global circulation, diffusive mixing, dry and moist convection, storms, and vortices). Species participating in ice and liquid cloud condensation (CH_4_, H_2_S, NH_3_, and H_2_O) are most sensitive to these processes.

Atmospheric abundances of disequilibrium species like CO and PH_3_ are some of the most challenging to interpret, but important for their potential to constrain the deep oxygen abundance. These species are linked to planetary elemental abundances by the interplay between quenched thermochemistry and mixing (Fouchet et al. 2009; Moses et al. 2020), which may vary spatially (Wang et al. 2015, see Fig. 2). Simultaneous measurements of multiple disequilibrium species are needed to break degeneracies between deep abundances and deep mixing efficiency (Wang et al. 2016; Giles et al. 2017). Remote sensing measurements of these species are particularly challenging. For example, CO is measured at low concentrations, and there is a degeneracy between stratospheric and tropospheric concentrations in spectroscopic retrievals, complicated by the externally-supplied oxygen from H_2_O. Retrievals of PH_3_ reach only shallow levels in the tropospheres of Jupiter and Saturn, with only upper limits available for Uranus and Neptune (Encrenaz et al. 1996, 2004), but at these levels, both condensation and UV photolysis act as loss processes of PH_3_. Multiprobe data provide a compelling opportunity to constrain both the concentrations of disequilibrium species at deeper levels in the troposphere, as well as their horizontal variation on the planet.

Strategic Research in the decadal survey calls for constraining “chemical processes, vertical mixing, and dynamical transport in all four giant planets by simultaneously measuring multiple tracers (e.g., temperature, condensable and disequilibrium species) over varied temporal, vertical, and horizontal scales, from... in situ measurements at Saturn, Uranus, and Neptune.”

Observations of the spatial/temporal variability of major chemical species—water in Jupiter, ammonia in Jupiter and Saturn, methane and H_2_S in Uranus and Neptune—demonstrate that mixing is incomplete, perhaps counteracted by moist convective storm precipitation (Guillot et al. 2020; Li et al. 2023). Measuring simultaneous vertical profiles of temperature and gas concentrations (CH_4_ and H_2_S) that trace convective processes on Uranus will lead to significant advances in our understanding of the convective process itself (Q7.3), and how it relates to observable phenomena such as storm activity, banded structures in the atmosphere (Fletcher et al. 2020), and unique polar regions. The convective process is also important due to its control over the long-term thermal evolution of the planet (Q7.2), particularly in comparison to Neptune, whose internal luminosity exceeds Uranus’ for reasons that are still unclear (Pearl et al. 1990; Pearl and Conrath 1991; Smith and Gierasch 1995; Kurosaki and Ikoma 2017; Friedson and Gonzales 2017; Markham and Stevenson 2021). Common processes are likely at work in multiple volatile condensation systems in the giant planet atmospheres, but for Uranus, the accessibility of the methane condensation region (and potentially the hydrogen sulfide condensation region) means that probe data could allow an entire condensation layer to be profiled. The results could then be applied to improve our understanding of other layers that are more difficult (or impossible) to observe, such as the water condensation region. Decadal survey Strategic Research in these areas includes constraining “the rate of heat transport in Jupiter, Saturn, Uranus, and Neptune by measuring thermal balance and vertical temperature profiles,” an activity well suited to secondary probe experiments since temperature profiles are spatially variable. The quest to understand how cloud-top color “ties to transport and chemistry in the atmospheres of Saturn, Uranus, and Neptune from in situ sampling of composition” would benefit from combined remote sensing of spatial variability, with detailed probe characterization of composition in multiple locations.

The composition of giant planet atmospheres is also influenced by dynamic interactions with their environments, particularly the exogenic delivery of volatile and non-volatile materials through ring rain, large impacts, and micrometeoroids (Q7.5, see for example Luszcz-Cook and de Pater 2013; Moses and Poppe 2017). The stratospheric abundance of species such as CS and CO have been taken as signs of geologically recent (within the past 1000 years) large impacts on Uranus and Neptune (Cavalié et al. 2014; Moreno et al. 2017). Probe measurements in the troposphere may not directly address this topic, due to the fact that slower stratospheric mixing timescales allow impact-related compositional anomalies to last much longer. But probe measurements of tropospheric species such as CO are important for reducing model-dependent uncertainties in stratospheric abundances (Luszcz-Cook and de Pater 2013). Improving our understanding of impact history at Uranus contributes to the Supportive Activity in Q4 of establishing a solar system chronology “through improved cataloging of impactor reservoirs... [and] more complete observations of present-day small body impacts in different contexts.”

## Spatial Variation in the Uranus Atmosphere

Spatial variation is the variation in longitude and latitude across the planet. The flagship probe would sample the vertical variation at a single point on the planet, but to achieve any kind of spatial sampling, multiple probes are needed.

Voyager 2 made the only spacecraft close-encounter with Uranus, measuring Uranus’ atmospheric temperature and compositional structure using radio occultation during egress. This signal was analyzed to determine the integrated path difference caused by refractivity variations through the atmosphere (Lindal et al. 1987). In order to invert this integrated path difference into an atmospheric structure model, one must make assumptions. The refractivity of a gas depends on its density, composition, and temperature.

We have a relatively small amount of data from Uranus compared to the other planets of the solar system, but many different forms of spatial variation have been observed. This includes variations in the temperature, composition, clouds and hazes. These are thought to be caused by different mechanisms but it is clear that the atmosphere of this planet is highly dynamic. This activity varies over different time scales that are still not well understood.

Due to the likely spatial variations in Uranus’ structure, as well as possible stochasticity in both space and time, multiple entry probe sites are preferable to properly contextualize spacecraft measurements.

### Atmospheric Structure

The Voyager 2 radio occultation provided temperature sounding to the 2.3-bar level, but required assumptions of hydrostatic equilibrium, a fixed relative humidity of methane above the cloud level, and a prescribed bulk mixing ratio of methane below the cloud level (Lindal et al. 1987). For a bulk methane-mixing ratio of 2.3%, the inversion gives a temperature of 101 K at the 2.3-bar level, but the range of temperatures spans some 16 K at this level for methane between 0–4% by volume. Therefore, entry probe measurements offer the only method to obtain unambiguous and non-degenerate measurements of temperature.

Multiple sources of spatial variation have been observed in the stratosphere (Roman et al. 2020; Rowe-Gurney et al. 2021). Evidence of a dynamic link between the troposphere and stratosphere has been observed, and understanding this link is important to understanding the planet’s temperature structure and chemical processes. Mid-infrared images from VLT-VISIR at 13 $\mu $m (Roman et al. 2020) revealed warm mid-latitude bands of acetylene emission in 2009 and hints of zonal variation with marginally greater emission at some longitudes. The observed distribution appears related and potentially coupled to the underlying tropospheric emission six scale heights below.

**Fig. 3:**
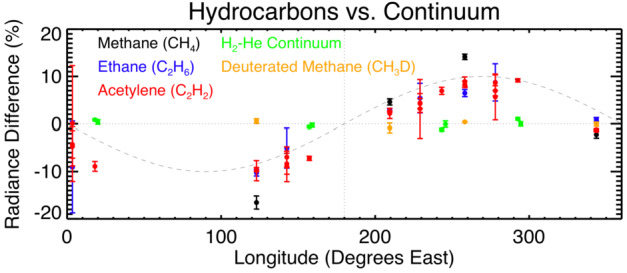
The percentage radiance difference from Uranus’ global average of chemical species across 360°of Longitude in 2007 from the Spitzer Space Telescope Infrared Spectrometer. Methane isotopologues, complex hydrocarbon species and the hydrogen-helium continuum are plotted (points with error bars) with a wavenumber 1 sinusoid for reference (dashed curve). Similar behavior in CH_4_, C_2_H_6_, and C_2_H_2_ suggests that temperature variation rather than composition drives the radiance enhancement, while lack of longitudinal variation in continuum and CH_3_D radiance may be due to sensitivity to levels deeper than the radiance anomaly. Adapted from Rowe-Gurney et al. (2021)

A variability of up to 15% in the thermal emission at stratospheric altitudes, sensitive to the hydrocarbon species at around the 0.1-mbar pressure level, was detected at a global scale at Uranus in 2007 using the Spitzer Space Telescope Infrared Spectrometer (Fig. 3, Rowe-Gurney et al. 2021). Optimal estimation retrievals show this is most likely caused by a change in temperature. Upwelling and adiabatic expansion might explain cooling of stratospheric temperatures and the activity in both spectral bands show that a few discrete cloud features exist at pressures less than 1 bar. These clouds show regions of condensation located high above the main cloud layers and likely indicate local perturbations in the temperatures or dynamics (from below). They could also influence the stratosphere, either by direct advection of mass, or by generating waves that propagate vertically, such as during Saturn’s 2010–2011 storm (Fletcher et al. 2012). The extraordinarily infrared-bright “beacon” in Saturn’s stratosphere, associated with the great storm in its troposphere, raises the possibility that tropospheric activity may also influence discrete stratospheric temperature anomalies on Uranus, but the picture is complicated because no beacon-like activity was observed in the near-infrared Keck images of Uranus, as was observed at Saturn (Sánchez-Lavega et al. 2019).

These instances of spatial variation are at different spatial scales and may originate from diverse features and processes. Uranus’ atmospheric structure may be time-dependent due to intermittency, as large storms may disrupt radiative-convective quasi-equilibrium (Smith and Gierasch 1995; de Pater et al. 2015; Markham and Stevenson 2018). This time variability also adds another dimension of complexity.

**Fig. 4:**
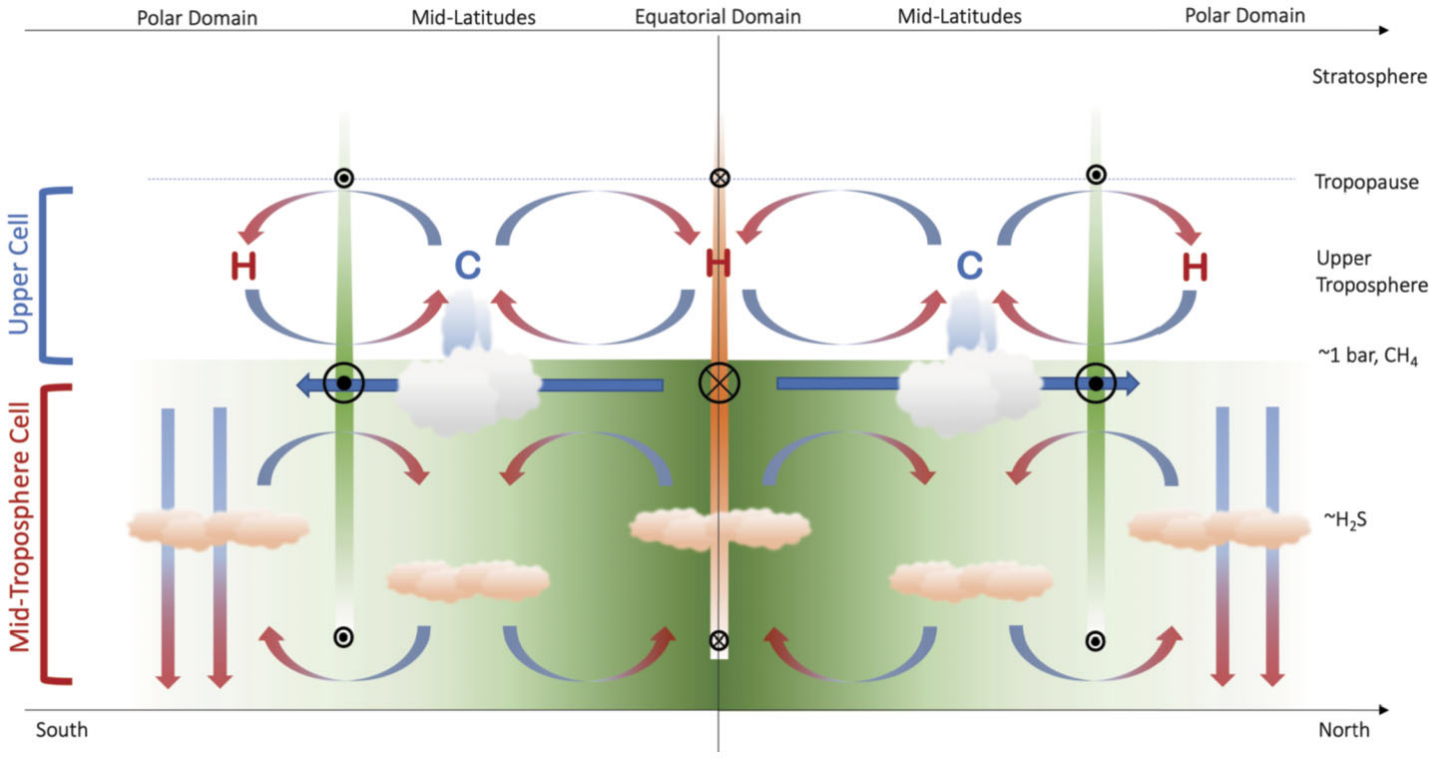
Schematic of the potential circulation in the troposphere and stratosphere of Uranus. Mid-Troposphere Cell: Extends down to around 50 bar from the 1 bar CH_4_ condensation level. Retrograde winds are shown by orange bars and circles with crosses. Prograde winds are shown by green bars and circles with dots. Upper Cell: Layer between the tropopause and the CH_4_ condensation level. Tropospheric temperatures are denoted by ‘C’ and ‘H’ for cold and hot. From Fletcher et al. (2020)

The upper tropospheric temperatures on both planets derived from Voyager 2 show cool mid-latitudes in the 80–800 mbar range, contrasted with warm equator and poles (Flasar et al. 1987; Conrath et al. 1998). The temperature contrasts suggest rising motion with adiabatic cooling at mid-latitudes, accompanied by subsidence and adiabatic warming at the equator and poles (Fig. 4). The upwelling at low latitudes condenses into discrete methane cloud features. Dry air would then be transported poleward and descend, thus inhibiting methane condensation at high latitudes (Sromovsky et al. 2011). This scenario is broadly consistent with the recent “holistic” aerosol model for Uranus and Neptune (Irwin et al. 2022), which finds that aerosols near the 1-bar level are not dominated by methane ice. Rather, this cloud layer is a secondary effect of methane condensation, where the CH_4_ ice rapidly precipitates after formation, but leaves behind a stable layer where the residence time is longer for hydrocarbon hazes mixed down from the stratosphere. Widescale upwelling would sustain the stable layer and help to suspend haze particles, while widescale downwelling would suppress formation of the stable layer.

### Composition

Characterizing the three-dimensional distribution of atmospheric constituents on Uranus is necessary in order to fully grasp how various chemical and physical processes are affecting said composition, and how the composition relates to the large-scale motion of the atmosphere (Orton et al. 2015). To understand the atmospheric and temperature structures discussed above requires characterizing the sources of opacity, and hence composition.

The Voyager 2 radio-occultation data is consistent with a layer of static stability caused by the larger molecular weight of methane relative to hydrogen (Lindal et al. 1987; Guillot 1995). Based on our experience with Jupiter (Li et al. 2017) and fluid dynamical arguments (Markham et al. 2023), there is no guarantee that methane should be well-mixed below the cloud level. Additionally, methane may follow compositional gradients arising from meridional circulation (Sromovsky et al. 2011).

**Fig. 5:**
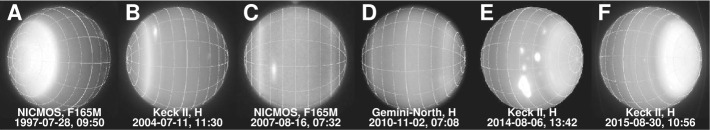
Global-scale variation in haze and methane concentration produces a bright polar cap over the sunlit polar regions of Uranus (Karkoschka and Tomasko 2009; Toledo et al. 2018; Sromovsky et al. 2019b; James et al. 2023), as seen in a series of H-band (1.6-μm) images from 1997 through 2015. The polar cap feature swaps hemispheres before and after the equinox. Figure from Sromovsky et al. (2019b)

Re-analysis of the Voyager 2 radio occultation data of Uranus in more recent years, combined with comparison to HST/STIS data, revealed a suspected methane depletion toward the poles (Sromovsky et al. 2011). Both Uranus and Neptune show this polar depletion of methane at their south poles in the NIR spectrum from Hubble (Karkoschka and Tomasko 2009, 2011). The intensity of this methane depletion is highly dependent on season and varies on multi-year timescales near the equinox (Fig. 5). With the next Uranian equinox in 2050, a proposed flagship mission will likely coincide with the rapid evolution of this polar cap feature.

**Fig. 6:**
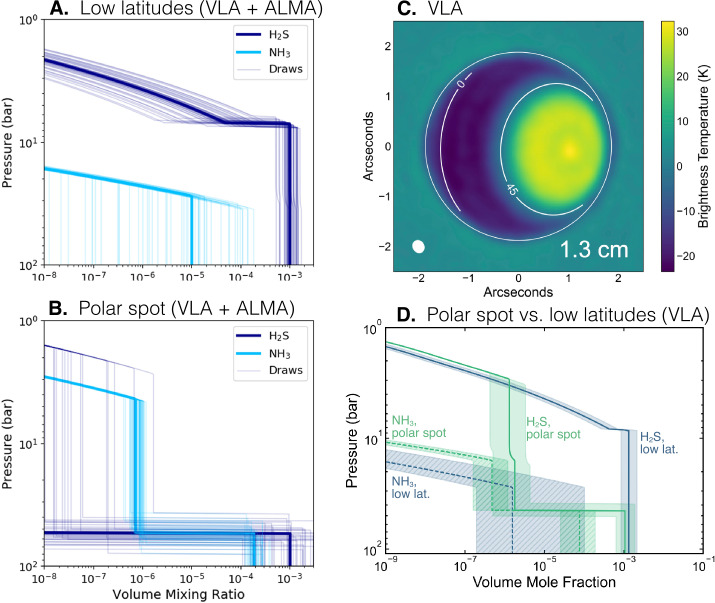
The polar compositional anomaly at Uranus extends to tens of bars. (A.,B.) Analysis of VLA + ALMA data by Molter et al. (2021) found a H_2_S-dominated troposphere at low latitudes and an NH_3_-dominated troposphere in the polar regions. (C.,D.) Higher spatial resolution VLA observations were analyzed by Akins et al. (2023), who again found differences in the H_2_S/NH_3_ ratio between polar regions and low latitudes, but H_2_S/NH$_{3} >$ 1 in both regions

This same pattern has also been seen in millimeter observations sensitive primarily to hydrogen sulfide (H_2_S) gas (Tollefson et al. 2019; Molter et al. 2021; Akins et al. 2023). Hydrogen sulfide and ammonia in the troposphere have been observed to have very different polar and low latitude profiles (Fig. 6). Other UOP instruments could provide advances in our understanding of compositional spatial variation, for example MWR (Levin et al. 2023), but this technique likewise suffers from a fundamental degeneracy between temperature structure and composition (Li et al. 2020). H_2_S absorption features have recently been detected in the NIR (Irwin et al. 2018, 2019b), but the latitudinal distribution has already been shown to exhibit the same polar depletion and mid-latitude enhancement as can be seen in methane and the hydrocarbons (Irwin et al. 2019a).

Spatially-resolved ground-based imaging of Uranus in the mid-infrared has revealed enhanced emission from stratospheric acetylene at mid and high latitudes compared to that at the equator (Roman et al. 2020). These spatial differences were found to be consistent with either a 16-K latitudinal gradient in the stratospheric temperatures or a factor of 10 gradient in the stratospheric acetylene abundance, arguing in favor of the latter based on the vertical motions implied by complementary upper-tropospheric observations. Probe measurements constraining vertical transport in the troposphere at multiple locations (i.e., in polar regions and at low latitudes) would be of value in the interpretation of this type of stratospheric compositional anomaly.

### Convective Activity

The strongly supersolar enrichment of volatiles in Uranus (as implied by the observed CH_4_ enrichment) suggests complex temperature and compositional structures in the atmosphere. Remote sensing observations can only probe down to the few-bar-level because gas and cloud opacity and Rayleigh scattering limit the penetration any deeper (Hueso and Sánchez-Lavega 2019). These levels are too shallow to reach the base of the H_2_S cloud, or to detect clouds of NH_4_SH or H_2_O at all (Weidenschilling and Lewis 1973; Atreya and Romani 1985; Sánchez-Lavega et al. 2004; Atreya et al. 2020).

In the gas and ice giants, above a critical abundance of the condensing species, moist convection is inhibited by the weight of the condensables rather than favored by latent heat release. This inhibition requires a sufficiently high abundance of condensables. In the case of Uranus, methane is the condensable that is sufficient to inhibit convection (Guillot 1995; Friedson and Gonzales 2017; Leconte et al. 2017; Markham and Stevenson 2021) as warmer parcels of gas are weighed down by methane molecules that are heavy compared to hydrogen and helium. This means the planet provides an extremely interesting laboratory to understand convection in hydrogen atmospheres (Hueso et al. 2020).

Precisely how the possible inhibition of convection affects the atmospheric temperature structure is currently not well understood, and we must therefore be skeptical of any a priori model for atmospheric temperature or composition structure.

Furthermore, convective inhibition may give rise to intermittent massive meteorological events that produce a time-dependent atmospheric temperature structure (Sugiyama et al. 2014; Li and Ingersoll 2015; Markham and Stevenson 2021; Li et al. 2023). Both Uranus and Neptune have discrete cloud activity that is both episodic and continuous. Unlike Jupiter and Saturn, most large scale systems at the ice giants are episodic and relatively short lived, disappearing after a few years. Some features, like the “Berg” feature at Uranus (Sromovsky et al. 2015) are more continuous and long-lived.

**Fig. 7:**
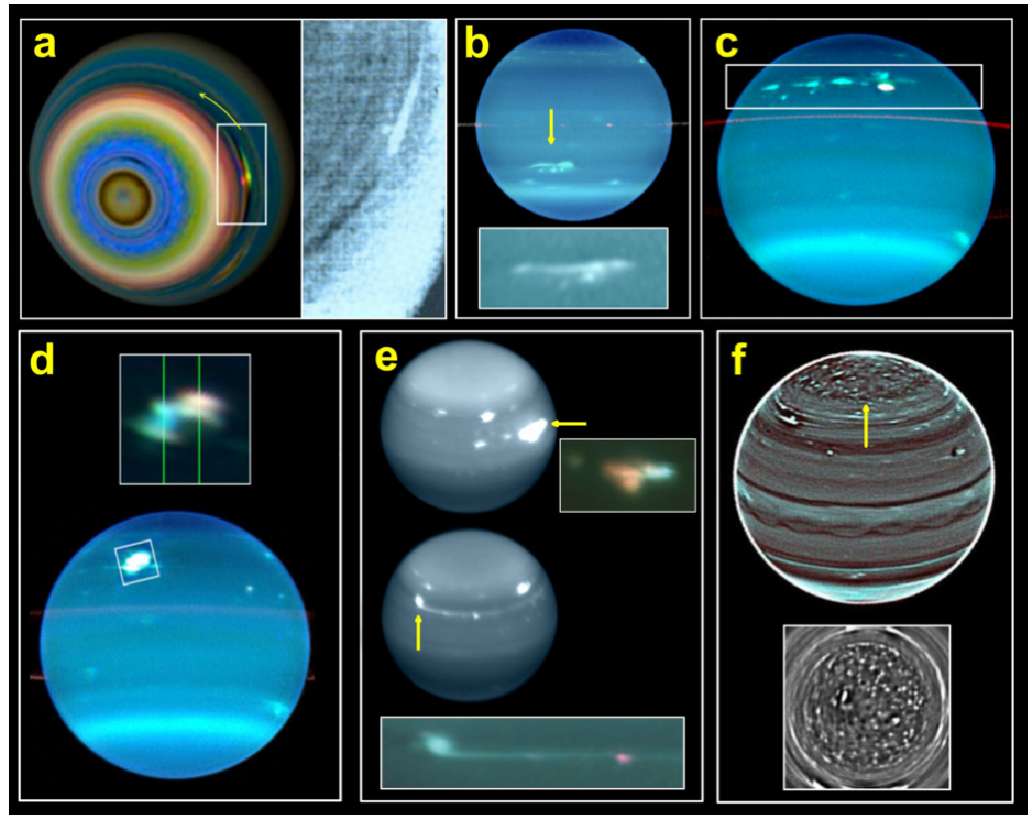
It is difficult to establish whether cloud features on Uranus are moist convective events or other phenomena. (a) An extended feature observed by Voyager 2 in 1986 in Uranus’ southern hemisphere, which could be produced by vertical upwelling in the presence of horizontal wind shear (Smith et al. 1986; Karkoschka 2015). (b) The “Berg” was a persistent feature with latitudinal drift and oscillations reminiscent of vortex behavior (Hammel et al. 2005; Sromovsky et al. 2019a; LeBeau et al. 2020), but no vortex rotation was directly resolved, and dramatic brightening events were interpreted as potential convective outbursts related to the feature (de Pater et al. 2011). (c,d) Approaching equinox, the region from 28°N to 42°N frequently generated bright cloud features reaching 300–500 mbar (Sromovsky et al. 2007; Sromovsky and Fry 2007). (e) Cloud activity in 2014 (de Pater et al. 2015) was interpreted as convective (Hueso et al. 2020), in part because a long aerosol trail was reminiscent of convective plume morphology seen on other giant planets (Sayanagi et al. 2013; Tollefson et al. 2017). But radiative transfer modeling showed that the extended trail was at a deeper level compared to the core of the feature, arguing against sheared plume-top interpretations (Irwin et al. 2017). (f) High-pass filtered imaging revealed banded patterns giving way above 60°N to a chaotic pattern of isolated compact features (Sromovsky et al. 2015), drawing comparisons to possibly convective “puffy clouds” in Saturn’s polar regions (Antuñano et al. 2018) as well as Jupiter’s high latitudes, where cloud structure is also different north of about 45°N accompanied by increased lightning frequency indicative of convection (Brown et al. 2018; Wong et al. 2023a). Figure from Hueso et al. (2020)

Uranus shows less discrete cloud activity than Neptune, though it does have some infrequent storms. Uranus’ meteorology was perceived to be relatively dormant during the Voyager 2 fly-by but has since then increased in activity as Uranus approached its northern spring equinox in 2007, as shown most prominently at near-infrared wavelengths. Episodic bright and dark features were observed in 2011 that were changing and moving over relatively short timescales (Sromovsky et al. 2012), and bright, long-lived cloud features have been observed multiple times (de Pater et al. 2011; Sromovsky et al. 2019a; Roman et al. 2018). One of the largest and brightest of these features was called the “Bright Northern Complex” (Fig. 7d), which attained its peak brightness in 2005 with clouds reaching pressures as low as 240 to 300 mbar (Sromovsky et al. 2007; Roman et al. 2018). In 2014 a similarly bright feature was observed in the near-infrared and estimated to reach to similar heights (de Pater et al. 2015). These features may be tied to vortex systems that exist in the upper troposphere, such as the prominent dark spot observed in 2006 at depths in the 1-4 bar pressure range (Hammel et al. 2009). This feature had bright cloud companions manifesting at lower pressures of around 220 mbars (Sromovsky and Fry 2005), which could be evidence of deep-seated features influencing the structure of the upper troposphere at certain longitudes.

The high methane abundance above the tropopause was historically the main argument in favor of moist convection in Neptune. The lower stratospheric methane concentration at Uranus may thus indicate a difference between the recent convective history in the atmospheres of the two planets. Evidence in favor of moist convective storms in Uranus (i.e. clouds formed by vertical ascending motions vertically transporting heat and powered in part by latent heat release) comes from observations of the cloud activity (Fig. 7). This is an incomplete source of information and shows a remarkable difference with what we know about convective storms in Jupiter and Saturn.

The physics of how planets with hydrogen atmospheres substantially enriched in heavy, condensing elements behave is of great interest for understanding exoplanets. Sub-Neptune/super-Earth class exoplanets, for example, may retain their heat for billions of years due to the inhibition of convection arising from the coexistence of hydrogen and silicate vapor (Markham et al. 2022; Misener and Schlichting 2022; Misener et al. 2023).

Because of the complex interplay between exotic meteorology, meridional circulation, and extant evidence of latitudinal variation in methane abundance, atmospheric probe measurements that can produce independent measurements of temperature and composition are essential to properly contextualize spacecraft observations.

Mean-zonal circulation is characterized on both ice giants by a broad retrograde tropospheric jet centered on the equator and prograde broad tropospheric jets in the mid-latitudes (Sromovsky and Fry 2005; Sromovsky et al. 2019a; Karkoschka 2015). The wind fields have none of the narrow, alternating structure (i.e. belts and zones) associated with Jupiter and Saturn. There is a banded structure at depth (i.e. below the hazes) that has been observed (Fig. 7f) but, unlike the two larger planets, there’s no notable connection between the winds and the bands (Karkoschka 2015; Sromovsky et al. 2015). For Uranus, the retrograde equatorial zone peaks at around 50 m/s. At both northern and southern mid-latitudes, a prograde jet blows at around 250 m/s, making it fairly symmetric between hemispheres.

Latitudinal variations in brightness, with maxima near the equator and south pole and minima at southern mid-latitudes, were observed at Uranus by Conrath et al. (1998) and again after reanalysis and comparison by Orton et al. (2015). This is consistent with a meridional circulation, with cold air rising at mid-latitudes and subsiding at both the poles and the equator (Fig. 4). The para-H_2_ fraction is at its minimum in areas of upwelling observed in the mid-latitudes yet at a much higher value in the high-latitude areas of the northern hemisphere that exhibited cooler temperatures Fletcher et al. (2020).

The role of moist convection and precipitation, its importance for determining the vertical structure of temperature, condensables and density, and the interplay of moist convection with the large-scale circulation are yet to be understood. Uranus possesses a cold atmosphere with abundant methane cloud activity that could be interpreted as convective, but the existing data does not allow us to determine which of the possible storm candidates observed are actually moist convective events. This methane condensation region is at a relatively low optical depth, and can be probed relatively easily. But without being able to distinguish between actively convective areas of the planet, we risk probing an anomalous region. This risk is significantly mitigated by deploying a multiprobe strategy.

The detection of radio signals from lightning at Uranus by Voyager 2 (Zarka and Pedersen 1986; Aplin et al. 2020) offers a way to characterize the deep convective activity. The Voyager observations were not localized. Measurements on an atmospheric probe could detect potentially more powerful signals trapped inside the ionospheric wave guide (Sect. 5.3), with measurements at different locations on the planet providing new constraints on the spatial distribution of deep convective activity.

## Secondary Probes at the Other Giant Planets

Of the giant planets, only Jupiter has been visited by an atmospheric entry probe. In the years following the Galileo Probe experiment, interest in returning with multiple probes was high (Sect. 1). Even with the major advances in our understanding of Jupiter’s atmosphere from Juno, the justification for a multiprobe experiment remains strong. The state of our current knowledge of the other giant planets also argues for multiple probes.

### Jupiter

The Galileo Probe’s science objectives included thermal and compositional measurements to at least 10 bar, with individual instruments (including the Galileo Probe Mass Spectrometer, GPMS) designed to operate to about 20 bar (Johnson et al. 1992; Niemann et al. 1992). The assumption of uniform mixing underpinned the rationale for the experiment, which was designed in part to determine Jupiter’s composition, including the bulk interior water abundance. This “well-mixed assumption” was based on theoretical models of chemical equilibrium cloud structure (Weidenschilling and Lewis 1973; Atreya and Romani 1985; Wong et al. 2015), but pre-Galileo signs that the assumption might not hold were given by infrared spectroscopic data and convective theory (Bjoraker et al. 1986; Stoker 1986; Lunine and Hunten 1987; Guillot 1995). This is important for Uranus as well, which may also violate the well-mixed assumption.

**Fig. 8:**
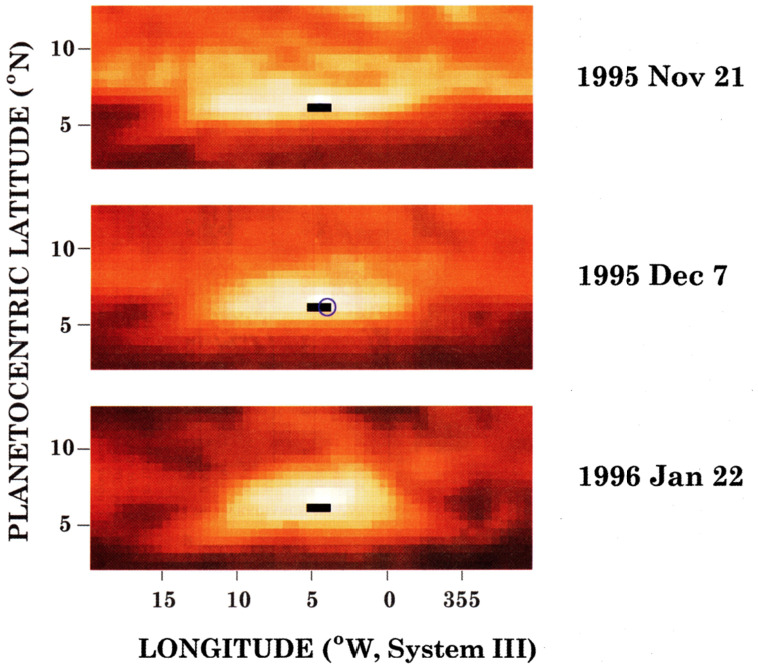
The Galileo Probe’s entry path on December 7, 1995 (black bar with terminal blue circle) lay within a 5-$\mu $m hot spot, whose morphology was interpolated from imaging data taken in November 1995 and January 1996. From Orton et al. (1998)

Probe entry into Jupiter’s atmosphere was constrained to happen close to the equator, due to requirements on entry angle, entry velocity, and ring-plane crossing radius (D’Amario et al. 1992). The targeted latitude of 6.6°N (planetocentric) placed the probe entry site at the right latitude to sample a “hot spot” of enhanced 5-$\mu $m emission (Fig. 8). In general, 5-$\mu $m hot spots owe their strong infrared brightness to simultaneous low column densities of cloud material and volatile absorbers NH_3_ and H_2_O (Bjoraker et al. 2022), and they are formed by an equatorially-trapped Rossby wave system (Ortiz et al. 1998; Showman and Ingersoll 1998; Showman and Dowling 2000; Friedson 2005).

Compositional profiles from the GPMS, Net Flux Radiometer (NFR), and probe-signal attenuation showed that all the cloud-forming volatiles—NH_3_, H_2_S, and H_2_O—were depleted at levels well beneath their equilibrium condensation levels (Niemann et al. 1996, 1998; Sromovsky et al. 1998; Folkner et al. 1998; Wong et al. 2004; Hanley et al. 2009). Still, the community entertained the possibility that the well-mixed assumption held at other locations on Jupiter, but that the probe’s entry into a 5-$\mu $m hot spot explained the deep volatile depletions found there (Atreya et al. 1997; Showman and Ingersoll 1998; Friedson 2005; Li et al. 2018). The well-mixed assumption could have immediately been discarded had there been a secondary Galileo probe at a different latitude. The validity of the assumption, even outside of hot spots, was already challenged by ground-based microwave observations of Jupiter, as well as by detailed comparison of the relative ratios of the volatiles in the probe site (de Pater et al. 2001; Wong et al. 2004, 2015; Wong 2009). But widespread abandonment of the well-mixed assumption would not be achieved until results from the Juno mission were unveiled.

**Fig. 9:**
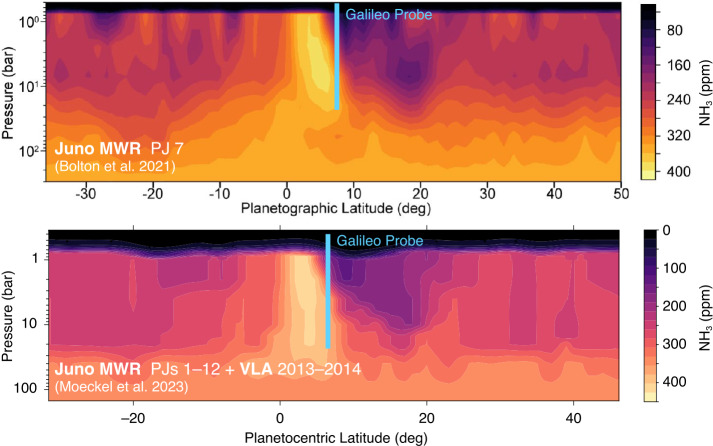
The Galileo Probe (blue bars) sampled Jupiter’s atmosphere at the edge of the anomalous ammonia-rich equatorial band. Ammonia concentrations in this region inexplicably exceed the deep well-mixed ammonia abundance. Adapted from Bolton et al. (2021), Moeckel et al. (2023)

Observations with the Juno Microwave Radiometer (MWR, Janssen et al. 2017) showed that on a global basis, ammonia is not well mixed until somewhere in the 20–100 bar range, a finding confirmed by spatially resolved VLA and ALMA observations (Bolton et al. 2017; Li et al. 2017; de Pater et al. 2019b,a; Moeckel et al. 2023). Figure 9 shows the deep ammonia depletion as retrieved in two independent analyses. Although it is now clear that disagreement between probe results and the well-mixed assumption is not simply an effect of the probe entry location in a 5-$\mu $m hot spot, the deep ammonia maps reveal that the Galileo Probe data were affected by proximity to another localized anomaly not recognized at the time: the high-NH_3_ equatorial band.

There is currently no explanation for the band of high NH_3_ concentration encircling Jupiter’s equatorial region (inside 0°–8°N, planetographic). The compositional anomaly extends from less than 1 bar to as deep as 20 bar, and concentrations within this band exceed the deep well-mixed ammonia abundance at all latitudes. Concentrations within the high-ammonia band exceed those at deeper levels below 20 bar, forming a compositional inversion. The Galileo Probe latitude (blue bars in Fig. 9) intersected the northern edge of the high-NH_3_ equatorial band, potentially explaining how the probe measured ammonia concentrations that exceed the deep well-mixed abundance derived from microwave remote sensing. A secondary probe measurement at a latitude well removed from the high-NH_3_ band would immediately reveal whether the lower ammonia abundance from microwave remote sensing (compared to high ammonia from the probe data) is an effect of the equatorial anomaly, or due to a systematic difference between probe data interpretation and microwave data interpretation. Because the highest ammonia concentration values were also based on microwave data—the attenuation of the probe carrier signal (Folkner et al. 1998; Hanley et al. 2009)—it seems likely that spatial variation is the largest factor in the disagreement between probe ammonia abundances and microwave remote sensing ammonia abundances. Multiprobes are ideal for comprehensive investigation of spatial variation.

The high-NH_3_ band has also been recognized as an opportunity to mitigate the degeneracy between temperature profile and absorber profile that affects microwave retrievals. Li et al. (2020) argued that the temperature profile is closer to a moist adiabat within the high-NH_3_ band, allowing for a retrieval of the water vapor concentration in that location from its subtle limb-darkening effect (Janssen et al. 2005). In other regions, the tropospheric temperature profile may be more uncertain; a range of observations and models suggest that Jupiter’s atmosphere is stably stratified, or subadiabatic (Wong et al. 2011, and references therein). The newest analysis of Juno MWR data by Li et al. (2022) allowed both temperature and ammonia to vary, by modeling deviations from the global mean state and including the effects of alkali metal opacity in the lowest-frequency channel of the instrument (Bhattacharya et al. 2023). This new analysis indeed finds subadiabatic temperature gradients on Jupiter, but not in the equatorial region, where a superadiabatic gradient was found. Superadiabatic gradients are unstable to convection, so Li et al. (2022) invoke the presence of a compensating water vapor gradient. The scenario is plausible, given the suggestion that the Galileo Probe encountered a superadiabatic gradient near 10 bar that may have been stabilized by a molecular weight gradient (Magalhães et al. 2002). Mysteries abound, because the mechanism for forming and maintaining the positive ammonia gradient (concentration increasing with altitude) at the base of the high-ammonia band is unknown, and this mechanism must also explain a negative water mixing ratio gradient in the same location, to stabilize the superadiabaticity. Given the degeneracy between temperature and compositional effects on microwave emission, simultaneous measurements of these quantities at multiple locations would provide valuable reference points to improve the fidelity of remote sensing inversions.

Although Juno is providing constraints on the water abundance (Li et al. 2020, 2022), it seems that the Juno observations will not be sufficient to construct a map of the deep H_2_O volume mixing ratio similar to the results available for ammonia (Fig. 9). The other condensable volatile, H_2_S, has only been detected by the Galileo Probe and has not been measured from remote sensing (Niemann et al. 1998; Wong et al. 2004). We are left with a whole suite of questions that would be closer to their answers if simultaneous composition and temperature measurements at Jupiter were available at multiple latitudes: Do all the volatiles have the same deep depletion as ammonia, or do they follow independent profiles? How is deep depletion created and maintained? What is the nature of the high-NH_3_ equatorial band? How are moist convection and deep NH_3_ depletion linked (Guillot et al. 2020)? Given the higher frequency of lightning detections in belts as compared to zones (Little et al. 1999; Brown et al. 2018), why does the deep depletion apply at all latitudes?

### Saturn

Saturn has not been visited by an atmospheric entry probe, but a Saturn probe option has been listed in NASA New Frontiers AOs in 2016 and 2023, following the recommendation of Visions and Voyages 2011, itself informed by a presentation describing a Saturn probe architecture that could reach 40 bar (Colaprete et al. 2009). Saturn probe concepts have been proposed to European Space Agency (ESA) Cosmic Vision AOs (Mousis et al. 2016). Decadal survey priority science questions that are addressed by multiprobes (listed in Table 1) are for the most part addressed equally well by Saturn data as Uranus data. A full understanding of the origin and evolution of the giant planets will await in-situ measurements at all four solar system targets. Specific multiprobe science drivers for Saturn, presented in this section, demonstrate the type of comparative planetology that can be done with multiprobe data from multiple planets.

The moist convective process in hydrogen atmospheres is key to understanding composition and dynamics in the diverse giant planets (Sect. 3.3). The moist convective style in a hydrogen atmosphere may range from frequent weak convection, to episodic powerful storm eruptions, depending on whether volatile abundances exceed a critical mixing ratio for convective inhibition (Guillot 1995; Sugiyama et al. 2011, 2014; Li and Ingersoll 2015; Leconte et al. 2017; Markham and Stevenson 2021). With respect to water, Saturn would appear to exceed the critical mixing ratio, while Jupiter may not, because lightning traces moist convection on a continuing basis at Jupiter, while Saturn’s lightning has been detected only within large singular storms (Dyudina et al. 2013; Sayanagi et al. 2013).

**Fig. 10:**
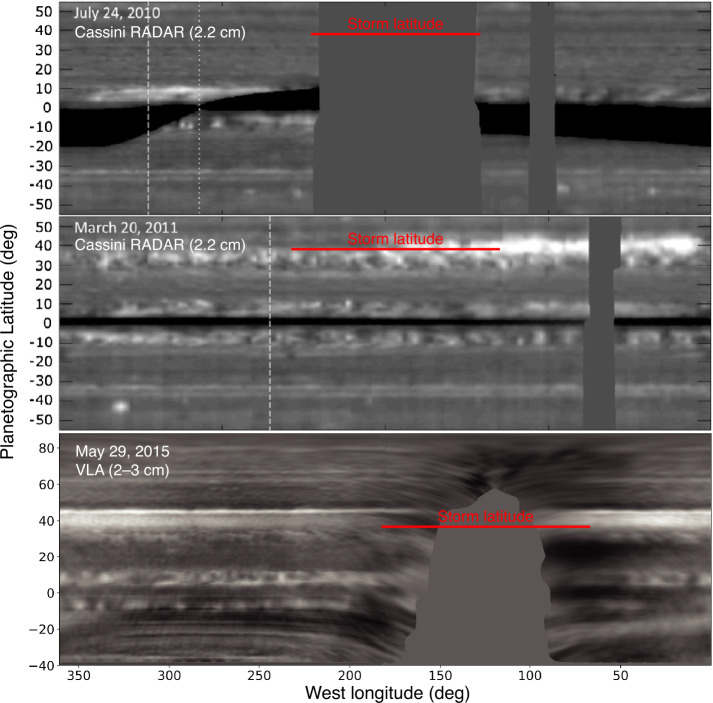
Saturn’s Great Storm erupted in 2010 and produced a long-term, planetary scale belt of high radio brightness temperature. The storm latitude of 38.2°N (Sayanagi et al. 2013) is marked in red. Adapted from Janssen et al. (2013), de Pater et al. (2023)

Measurements of conditions relating to water moist convection at Saturn may be directly comparable to measurements at Uranus of properties within the methane cloud (possibly exceeding the critical value for convective inhibition) and the H_2_S cloud (possibly below the threshold for convective inhibition). Data from multiple planets and cloud layers is essential for quantitatively testing our understanding of the convective process. Multiprobe measurements are particularly important because microwave observations of Saturn show multi-year changes in the ammonia distribution following the 2010 great storm (Fig. 10). Compositional anomalies in Saturn’s atmosphere may be long-term remnants of great storms dating back to the earliest known example in 1876 (Li et al. 2023). Understanding how compositional anomalies trace past convective outbursts at Saturn—where we have a good record of convective outbursts spanning more than a century—could be valuable for interpreting compositional profiles at the ice giants, where we do not have good constraints in the pre-Voyager/pre-Hubble era on the timescale or periodicity of activity (Smith and Gierasch 1995; Friedson and Gonzales 2017; Leconte et al. 2017; Li et al. 2023). The compositional anomalies on Saturn are localized, driving the need for probe measurements at multiple sites to obtain a full picture of how moist convection works in hydrogen atmospheres.

Compositional and thermal profiles both at the equator and at higher latitudes would also test the extent to which Saturn resembles Jupiter, with its high-NH_3_ equatorial band. The top two panels of Fig. 10 are from Cassini RADAR observations conducted with the spacecraft in orbit near the equatorial plane, such that interference from the ring system makes it difficult to ascertain a resemblance to Jupiter at the equator. The bottom panel was obtained from Earth at a high sub-observer latitude (29.1°N), so that ring artifacts can be seen in the southern hemisphere, but the equatorial region clearly shows low brightness temperature that may be indicative of ammonia enhancement similar to Jupiter.

### Neptune

The path toward multiprobe exploration of Neptune is not currently clear, but the same science drivers apply (Table 1). As with Uranus, Neptune seems to have a much higher NH_3_/H_2_S ratio in the polar regions than at lower latitudes (Tollefson et al. 2021), and methane also varies with latitude (Karkoschka and Tomasko 2011). Although there is some hope of measuring the methane abundance beneath the CH_4_ ice condensation level with entry probes at Uranus and Neptune, probes limited to 20 bar or so will not be able to measure bulk atmospheric mixing ratios of H_2_O, NH_3_, and probably H_2_S, especially considering the potential that some of these species may be dissolved into deep water cloud droplets. Nevertheless, measurements at multiple locations will help constrain the range of compositional variation and set lower limits on abundances.

With the next NASA flagship effort presumably focusing on Uranus, miniaturized probes may be the only option for in-situ sampling at Neptune. The same technologies that would enable compact secondary probes accompanying a larger primary probe would enable small probes to ride along on potential smaller missions to Neptune or beyond, perhaps as part of a future New Frontiers mission class. Neptune may be reachable in a cruise time of 10–15 years with nuclear propulsion, as discussed in a Chinese mission concept that lacked an atmospheric probe (Yu et al. 2021; McCarty et al. 2022). A miniaturized probe would be easier and less costly addition to such a mission (compared to a flagship-class probe), enabling the mission to address many of the Table 1 science questions.

## Key Measurements for Secondary Probes

Based on the discussion of science drivers for Uranus multiprobe exploration (Sect. 2), our current knowledge of spatial variation at Uranus (Sect. 3), and our experience and knowledge of the other giant planets (Sect. 4), the core measurements from secondary probes are the atmospheric structure, vertical profiles of species whose concentrations vary horizontally, and vertical wind shear. Table 2 links specific measurement goals to the themes of planetary origins and dynamic processes (see Table 1), and it lists candidate science instruments that could conduct the measurements.

**Table 2:** Secondary/Multiprobe Measurements

Instrument	Measurement goals
*Theme: Origins (Q1, Q2 in Table* *1**)*	
ASI^a^	Measure profiles of temperature and pressure (and density and sound speed if possible) to determine the static stability.
	Determine whether heat is transported by convection or radiation.
CS^b^	Determine the maximum concentration along the descent path of volatile species such as CH_4_, NH_3_, H_2_S, H_2_O.
	Determine the concentration of disequilibrium species such as CO and PH_3_.
micro-TLS^c^	Determine the isotope ratios of C, H, O, N, and S in atmospheric molecules.
	Determine the maximum concentration along the descent path of volatile species such as CH_4_, NH_3_, H_2_S, H_2_O.
	Determine the concentration of disequilibrium species such as CO and PH_3_.
*Theme: Dynamic processes (Q7 in Table* *1**)*	
ASI	Measure profiles of temperature and pressure (and density and sound speed if possible) to determine the static stability and mode of vertical heat transport.
	Measure simultaneous profiles of temperature and composition to help break degeneracies in spatially resolved remote sensing retrievals.
	Measure the ortho/para hydrogen ratio to determine static stability and trace the mixing history.
CS	Determine vertical variation along the descent path of volatile species such as CH_4_, NH_3_, H_2_S, H_2_O.
	Determine whether the concentration of disequilibrium species such as CO and PH_3_ varies horizontally compared with other probe measurements.
micro-TLS	Determine vertical variation along the descent path of volatile species such as CH_4_, NH_3_, H_2_S, H_2_O.
	Determine whether the concentration of disequilibrium species such as CO and PH_3_ varies horizontally compared with other probe measurements.
USO^d^	Measure profile of horizontal wind speed as a function of depth.
Lightning^e^	Detect deep moist convection via radio emissions from remote lightning.

^a^Atmospheric Structure Instrument. Measures ambient temperature and pressure during descent

^b^Chemiresistive Sensor. Measures partial pressure of reactive gas species with technologies such as field-effect transistors (FET) with doped nanomaterials (Li et al. 2003; Hannon et al. 2016; Fahad et al. 2017; Sultana 2020)

^c^micro Tunable Laser Spectrometer. Measures infrared spectral line absorption to derive relative abundances and isotope ratios of molecules (Webster et al. 2023)

^d^Ultra Stable Oscillator. Enables wind speed determination from measurement of carrier signal Doppler shift

^e^Lightning detector. Antenna and receiver package for detection of signals in VLF (3–30 kHz) range

Additional instrument options could make measurements of spatially variable quantities, but these are not listed in our core discussion because their links to both origins and dynamic process priority science questions were considered significant but not as comprehensive. These include net flux radiometer experiments (Apéstigue et al. 2023) or nephelometers (Banfield et al. 2005). For a mission where a miniaturized probe can be accommodated, but there is no primary flagship-class probe, some of these additional instruments should be considered.

### Atmospheric Structure

The most crucial dual measurements for a secondary probe will be the temperature and pressure structure. This measurement is in the scope of the “Atmospheric Structure Instrument” (ASI), a package which combines individual sensors for pressure and temperature measurements. The measurements of pressure and temperature alone provide a powerful constraint which, when combined with remote sensing data and theory, provide far less model-dependent results for the atmospheric convective state and compositional structure. Such a measurement would allow for a far more powerful assessment of Uranus’ dynamical state (Q7.2 in Origins, Worlds, and Life 2022). Providing ground-truth dramatically reduces the degeneracies of remote sensing alone discussed in Sect. 3.

Additional ASI capabilities would be valuable because atmospheric structure is still not fully characterized by measurements of only the two basic thermodynamic quantities of pressure and temperature. The most obvious, and likely most useful supporting measurement would be of density. An independent density measurement provides two key pieces of information: the mean molecular weight using the ideal gas equation of state, and the vertical spatial structure of the atmosphere by assuming hydrostatic equilibrium. The former can be used as a proxy for changes in composition, discussed further in Sect. 5.2. The latter can be used to more precisely constrain the relationship between pressure level and optical depth for remote sensing.

Sound speed measurements would be of similar usefulness. Independent measurements of density, pressure, and sound speed uniquely specify the Grüneisen parameter $\gamma $ from the adiabatic relationship

1 $$ c_{s}^{2} = \frac{\gamma p}{\rho}, $$

and by extension the specific heat capacity through the relationship

2 $$ c_{p} = \frac{\gamma}{\gamma - 1} R, $$

relevant to an ideal gas. These quantities can aid in constraining the relative abundances of ortho- and para-hydrogen (Banfield et al. 2005), relevant to atmospheric dynamics, as well as further constrain the compositional structure. Additionally, the heat capacity of an ideal gas atmosphere combined with gravity define the dry adiabatic lapse rate

3 $$ \Gamma _{\mathrm{ad}} = -\left .\frac{dT}{dz}\right \rvert _{\mathrm{ad}} = - \frac{g}{c_{p}}, $$

allowing one to explicitly detect regions of subadiabaticity and superadiabaticity, distinguishing moist convective regions and regions of static stability.

**Fig. 11:**
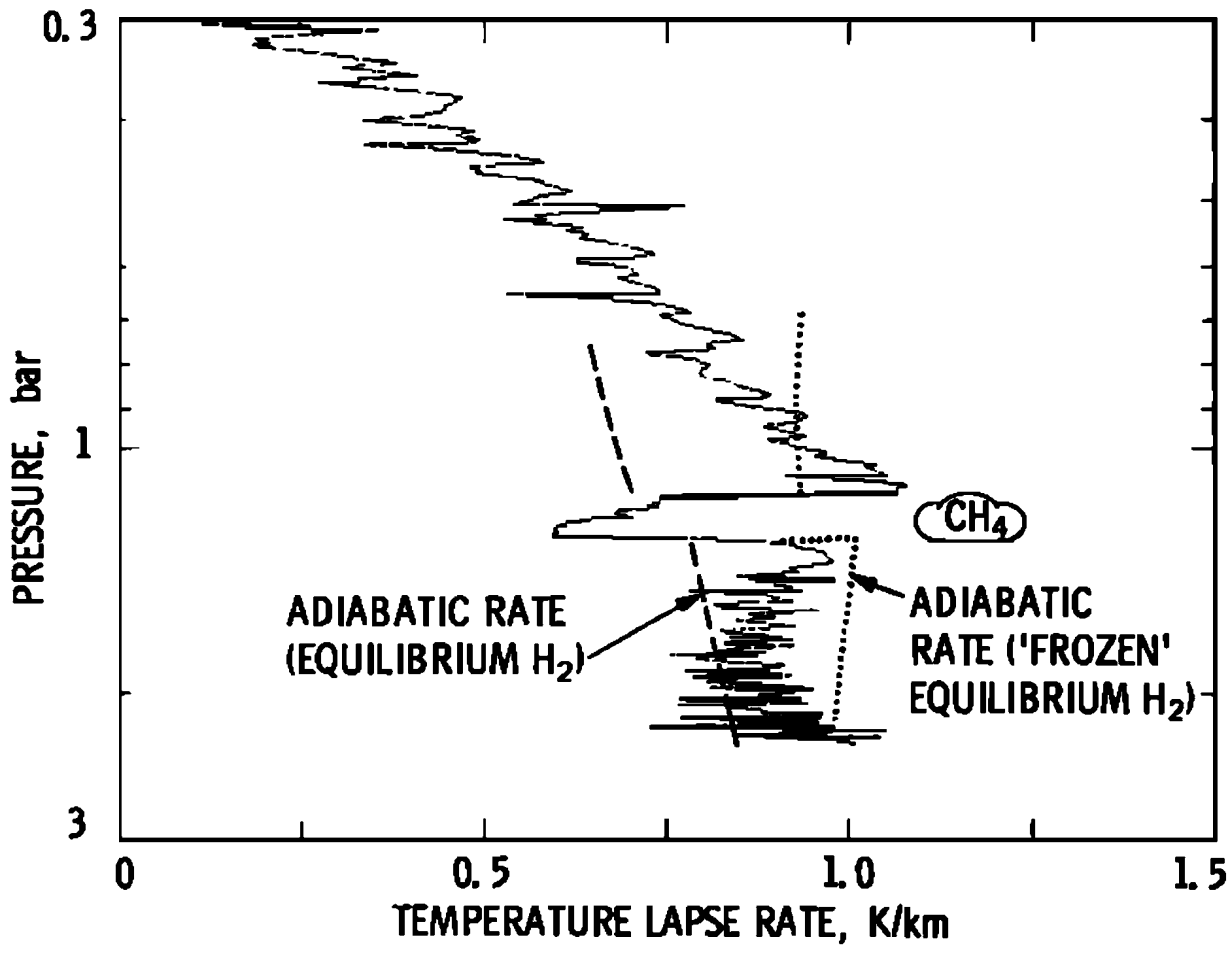
An anomaly near 1.2 bar in the Voyager 2 radio occultation data may include a superadiabatic layer above the cloud top, compensated by a molecular weight gradient. Uncertainties in the actual static stability come from degeneracies between temperature and density in the radio occultation data. Figure from Lindal et al. (1987)

For all measurements, a resolution of about 10% of the vertical scale height would be necessary in order to resolve features such as the “Lindal blip” from Fig. 11. This region is key to properly characterizing the atmosphere, and corresponds to the methane cloud level. It has been interpreted as either the simple base of the cloud layer (Lindal et al. 1987), or possibly evidence of static stability due to the inhibition of convection (Guillot 1995). The latter interpretation is supported by Irwin et al. (2022), who find that aerosols in this layer are too absorbing to be methane ice itself, and may represent haze particles that remain suspended due to weaker mixing in the stable layer.

### Composition

While a flagship-class primary probe would be responsible for a broad compositional survey using mass spectrometry, a secondary probe has the potential to provide in-situ constraints on latitudinal compositional gradients. Trace species, especially out-of-equilibrium species and products of photolysis, may vary latitudinally throughout Uranus due to differences in insolation, meridional circulation, and convection. While a detailed inventory of these variations would be of interest, it is likely more practical to focus more narrowly on more abundant species. Of particular interest are CH_4_ and CO. Methane, with its high abundance, is expected to condense between 1 and 2 bars on Uranus. As summarized in Sect. 3, the dynamical nature of methane moist convection is poorly understood. Due to the degeneracy between composition and temperature gradients in many remote sensing techniques (Figs. 3, 6, 9, 11, and relevant discussions), unambiguously determining whether regions of static stability exist will likely require ground truth.

Beyond atmospheric dynamics, a secondary probe would offer possible hints about Uranus’ interior structure and formation history. While precision measurements of the gravity field provide some constraints on the density profile of the planet’s interior, this information alone cannot uniquely specify composition for a planetary interior likely composed of a mixture of rock, ice, and gas (e.g., Teanby et al. 2020; Movshovitz and Fortney 2022; Neuenschwander and Helled 2022). Measurements of species in the envelope could be diagnostic of composition at depth. For example, a determination of the ratio of carbon to nitrogen in the envelope could elucidate the thermodynamic state and composition of the envelope-mantle interface when combined with simulations or laboratory information about the relative partition of ammonia and methane between a coexisting gas-rich and water-rich environment. The relative abundances of species carrying C, S, and N are spatially variable in the atmosphere, so improved knowledge of this variation from spatially distributed in-situ sampling provides better constraints on the corresponding bulk relative abundances in the envelope.

Because compositional variations are likely to be dominated by variations of CH_4_ concentration (particularly at $p \lesssim $ 5 bar), measurements of density alone would already provide a useful constraint as discussed in Sect. 5.1. However, greater precision and information about other condensing species at greater depth requires a method to measure these constituents directly. Performing this measurement with a traditional mass spectrometer on a secondary probe would likely exceed limits on cost, volume, and mass, but alternative technologies could enable such measurements. Two examples are chemiresistive sensors and miniaturized tunable laser spectrometers (CS and micro-TLS, see Table 2). Chemiresistive sensors are chip-scale devices that detect gas species by changes in resistivity, often using 1D and 2D nanomaterials doped typically with metal oxides to increase sensitivity and/or specificity. This class of sensor is used across a growing range of industrial and medical applications, and is now being adapted to planetary exploration (Sultana 2020). A tunable laser spectrometer has successfully been used at Mars, and research is ongoing to miniaturize the technology to the point where it could potentially be carried on a small secondary probe (Webster et al. 2023).

The highest priority targets for these composition sensors are CH_4_, CO, H_2_S, and NH_3_. Each species is expected to exist in abundances on the order of a tenth of a percent or more (e.g., Hueso and Sánchez-Lavega 2019). At the 10-bar level, H_2_O would be detectable if it is close to its saturated volume mixing ratio of about 0.05%. So far above the cloud base, such a measurement would be valuable for isotopic measurement or characterization of spatial variability, but not as a constraint on the bulk oxygen abundance. To make useful statements about spatial variations and elemental ratios, measurements of condensing species should be made to about 10% accuracy.

Although non-condensing, CO is of interest because of its relevance to constraining the oxygen abundance of Uranus’ deep envelope, relevant to Q1.2., Q.2.2, Q7.1.; and the convective contact between the methane and water cloud levels relevant to Q7.2. Understanding the deep mixing efficiency needed to interpret CO in the context of deep bulk abundances would be advanced by the measurement of additional disequilibrium species such as PH_3_.

### Convective Activity

The convective state of the water cloud layer is likely to be difficult to probe directly due to its great depth, but theoretical studies suggest lightning activity due to water storms on Uranus may be significant (Aglyamov et al. 2023). A lightning detector onboard the primary and secondary probe could provide information about the strength, intermittency, and spatial variability of convection at the water cloud layer. Such observations could aid in constraining the deep water abundance, and understanding the heat flow in Uranus’ envelope as well as distinguishing between a convectively active or inhibited state. Targeting the VLF (3–30 kHz) frequency range would have the greatest value for lightning investigations conducted by a secondary probe, because emissions may be strongest in this range, and the probe’s location inside the ionospheric barrier would provide sensitivity to signals undetectable by spacecraft (Aplin et al. 2020).

Combined atmospheric structure and compositional measurements will allow for a better determination of the convective state of the atmosphere. An atmospheric profile that measures pressure, temperature, and volatile abundance can determine whether the atmosphere is undergoing quasi-equilibrium convection (as observed, for example, around the Earth’s tropics Emanuel 2007), a stably stratified structure in global radiative-convective equilibrium (as predicted by e.g., Leconte et al. 2017; Markham and Stevenson 2021), or if the atmosphere is susceptible to intermittent convective events (as observed in the Earth’s mid-latitudes). With these three variables, one can calculate the convective available potential energy (CAPE) and convective inhibition (CIN; e.g., Sankar and Palotai 2022). A measurement of vertical wind shear would likewise inform the propensity of the atmosphere to energetic storms by analogy to terrestrial meteorology (e.g., Draxl et al. 2014). Additionally, measurements of CO would provide information about the timescale of vertical motion from the water cloud level and the oxygen abundance of the envelope, containing information about the convective state of the atmosphere between these two dominant cloud levels by assessing the quench location of CO at depth (perhaps with additional information from measurements of complementary disequilibrium species such as PH_3_).

The notion of convective inhibition has so far been theoretically explored as a 1-dimensional phenomenon in numerous studies (Guillot 1995; Leconte et al. 2017; Friedson and Gonzales 2017), and across small domains in 2- and 3-dimensional simulations (Nakajima et al. 2000; Sugiyama et al. 2014; Li and Ingersoll 2015; Ge et al. 2022; Leconte et al. 2024). Measuring the spatial variability of convective inhibition would serve as an invaluable constraint on theoretical models of hydrogen convection in the presence of volatile phase transitions. Moreover if the probe can reach sufficient depth, comparing the behavior of the methane cloud deck to the H_2_S and NH_4_SH cloud decks would place further constraints on the sensitivity of the behavior of convective inhibition to volatile abundance, as linear theory predicts that while the methane cloud deck should be convectively inhibited, deeper cloud decks such as H_2_S may not be (Leconte et al. 2017). Therefore a probe expected to reach a depth of tens of bars would further benefit from instruments capable of measuring H_2_S and NH_3_ for the purpose of understanding atmospheric convection as well as composition at depth as described in Sect. 5.2. Probes sampling multiple locations could assess the degree to which convective inhibition may exist as a local vs. a global phenomenon.

## Opportunities and Challenges for Secondary Probes

### Secondary Probe Design Considerations

The scientific goal of secondary probes focuses on understanding the physical and chemical processes that shape and maintain the ice giant atmospheres by measuring quantities that change between entry locations. Because secondary probes target only the spatially variable quantities, they require only a subset of the instruments that are carried in a large main probe. Spatially variable quantities that are key to understanding the tropospheric circulation and energy transport include the distribution of cloud-forming and disequilibrium species, vertical stratification, and horizontal wind component. A secondary probe that focuses on spatially variable quantities could rely on more miniaturized technologies and weigh much less than a large probe carrying a mass spectrometer. Table 3 compares past probe designs to highlight two points: first, across different probe designs, the instrument mass fraction tends to be between 10–15%; and second, a mass spectrometer takes up a major portion of the instrument mass.

**Table 3:** Comparison of planetary entry probe designs.

Missions	Total Mass	Entry System	Descent Module			
	CBE^a^+Margin	Aeroshell+Chutes	Instrument Total	Mass Spectrometer	Other Instruments	Battery
Galileo Probe^b^	335 kg	219 kg (65%)	35 kg (10%)	13.2 kg^g^	21.8 kg	7.5 kg (Li-SO_2_, 2.2%)
Huygens^c^	318 kg	118 kg (37%)	48 kg (15%)	17.3 kg^h^ +6.1 kg pyrolizer^i^	24.2 kg	13 kg (Li-SO_2_, 4%)
2010 Uranus Study^d^	127 kg	41 kg (32%)	17 kg (13%)	9.2 kg	7.9 kg	11.3 kg (Li-SOCl_2_, 9%)
2017 IG SDT^e^	321 kg	147 kg (46%)	33 kg (10%)	17 kg	15 kg	17 kg (Li-Ion, 5.3%)
2020 SNAP^f^	30 kg	15 kg (50%)	6.6 kg (22%)	N/A	6.6 kg	0.34 kg (Li/CF_*x*_, 1%)
2021 UOP^j^	268 kg	121 kg (45%)	22 kg (8%)	17.8 kg	4.1 kg	11 kg (Li-SOCl_2_, 4%)

^a^Current Best Estimate

^b^Some values from Johnson et al. (1992) were updated based on information from General Electric Re-entry Systems Operations (1984) and O’Neil (1990)

^c^Huygens Probe (Clausen et al. 2002)

^d^Planetary Science Decadal Survey 2013-2022 Uranus Mission Concept Study (Hubbard and Marley 2010)

^e^Ice Giants Science Definition Team report (Hofstadter and Simon 2017)

^f^Small Next-Generation Atmospheric Probe (Sayanagi et al. 2020)

^g^Galileo Probe Mass Spectrometer (GPMS) mass is from Niemann et al. (1992)

^h^Huygens Gas Chromatograph Mass Spectrometer (GCMS) mass is from Niemann et al. (2002)

^i^Pyrolyser mass is from Israel et al. (2002)

^j^Uranus Orbiter and Probe Decadal Study Report (Simon et al. 2021)

**Fig. 12:**
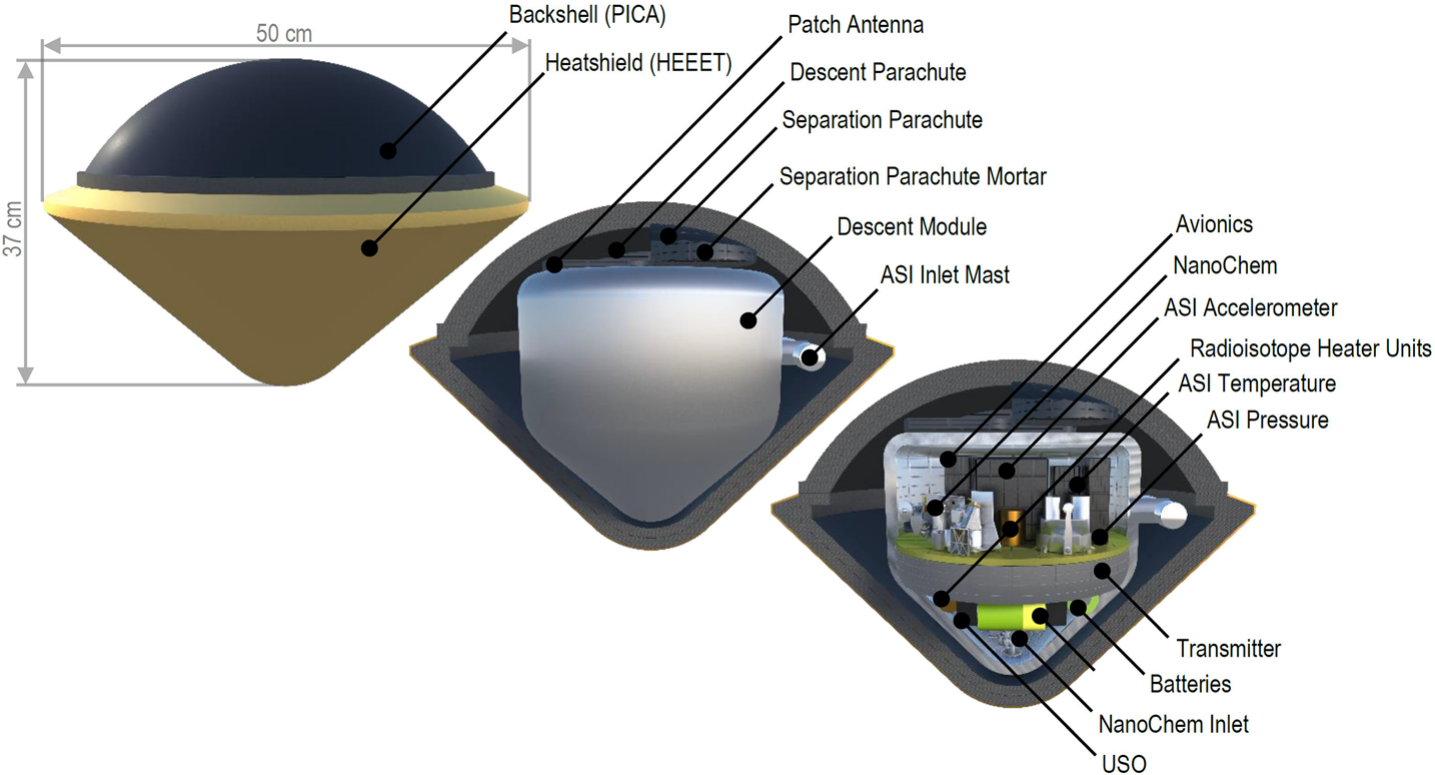
Design schematics of SNAP, from Sayanagi et al. (2020)

The Small Next-generation Atmospheric Probe (SNAP) study (Sayanagi et al. 2020) designed a 30-kg probe that focuses on spatially varying quantities. The SNAP concept’s science objectives are to determine (1) the vertical distribution of cloud-forming molecules; (2) thermal stratification (i.e. temperature and pressure as functions of altitude); and (3) horizontal component of the wind speed as a function of altitude. The first objective was based on a hypothetical chip-scale instrument that would measure vapor concentrations (see Sect. 6.4), while the second and third objectives were built upon well-established instrument heritage, namely the Atmospheric Structure Instrument (ASI) and the Ultra-Stable Oscillator (USO), respectively. The 30-kg SNAP mass estimate includes a thermal protection system (TPS) mass of 15 kg, which scales from the Galileo Probe TPS mass of 222 kg, considering that SNAP has a 6.5 times smaller aeroshell surface area, 23% of the Galileo Probe total heat load (Milos et al. 1999), twice the heat pulse duration compared to Galileo entry, and 70% the TPS density using HEEET instead of carbon phenolic (Venkatapathy et al. 2020). The SNAP design’s high 22% instrument mass fraction was enabled by a Li/CF_*x*_ battery with four times higher energy density than a Li-Ion battery (Krause et al. 2018). See Fig. 12 for the schematics of the SNAP design.

### Cost

Adding a second probe increases the complexity and cost of the mission; however, the SNAP study (Sayanagi et al. 2020) demonstrated that the cost of adding a small probe that targets spatial variabilities would be significantly less than a large planetary probe, and would increase the overall mission cost by a small fraction. The cost to add one SNAP to the orbiter is estimated to be about 80 million dollars in $FY2018. The $80M estimate includes the cost to design and build the probe, operational costs, modification necessary to the orbiter to mount SNAP, and a 30% reserve. While this estimate for a secondary probe cost is about twice as much as a large instrument (e.g., $38M for the thermal IR camera in the UOP study, Simon et al. 2021), it is significantly less than the $278M estimated cost of the primary probe hardware and a small fraction of the $2.8B estimated for the total mission cost (in $FY2025). Thus, the SNAP study demonstrated that the cost of adding a second probe to measure spatially variable quantities represents a relatively small fraction of the total mission cost.

A secondary probe could be incorporated into the UOP mission as either a directed component (like a facility instrument, a part of the core NASA mission design) or a competed component (available as an option for community proposals). Including the secondary probe as a directed component from the beginning of mission planning is advantageous because the need for radiogenic heating (Sect. 6.5) requires significant lead time for nuclear materials launch regulatory approval. Alternatively, the announcement of opportunity for competed instruments on the mission could include a secondary probe within its scope (Wong et al. 2023b).

### Trajectory

When the orbiter must be used to receive data transmitted from the probe, a major challenge in any probe mission is to design the trajectories so that the orbiter is within a communication range of the probe during the probe’s atmospheric descent. While the Huygens probe succeeded in returning data directly to Earth from the probe, such direct-to-Earth data transfer is likely unrealistic for any Uranus probe due to the long communication range. Thus, a multi-probe mission would necessarily add complexity to the orbiter trajectory in order to deliver the probes to well-separated entry locations and receive data from the probes.

In addition, a multi-probe mission may increase the propellant required for the spacecraft’s orbit insertion maneuver because the secondary probe(s) will likely need to stay attached during orbit insertion. In a single-probe mission, the probe can be released prior to orbit insertion to reduce the mass to be delivered in orbit. For example, the Galileo orbiter released the probe about 6 months prior to its Jupiter orbit insertion, and thus reduced the propellant need by not carrying the probe mass during the Jupiter orbit insertion maneuver. Recent multi-probe architecture studies (Sayanagi et al. 2020; Arora et al. 2021) illustrated the difficulties of releasing two or more probes before orbit insertion and subsequently placing the orbiter at a location to receive data from both probes entering separate locations. These issues are solved if the secondary probes are released from the orbiter during orbits subsequent to orbit insertion. Sayanagi et al. (2020) estimated that carrying a 30-kg probe and 4 kg of mounting hardware through the Uranus orbit insertion maneuver with a $\Delta V$ of 1680 m s^−1^ would consume 43 kg of additional propellant. The concern of additional propellant for orbit insertion prior to secondary probe release could be largely eliminated if the mission uses aerocapture for orbit insertion (Girija 2023), although a higher fidelity assessment is needed to understand the impacts of aerocapture on mission design, spacecraft design, and concept of operations.

After the orbit insertion, any secondary probe would need to be released at most one probe per orbit. To minimize the $\Delta V$ for the probe targeting maneuver for each probe, the probes should be released near the apoapsis from where the orbiter and the probe would follow roughly parallel trajectories, which should place the orbiter above the probe during the probe’s atmospheric descent to allow the orbiter to receive data from the probe. Initial capture orbits have a period of several months, so the probe must satisfy its power and thermal requirements for at least 30 days after being released from the orbiter, which raises challenges for heating and power (Sect. 6.5). Nevertheless, Sayanagi et al. (2020) and Arora et al. (2021) demonstrated that releasing secondary probes after orbit insertion is a viable strategy to deliver the secondary probes to different locations on Uranus.

### Instrument Maturity

Mature instrument options exist to address a minimum threshold set of science objectives to understand atmospheric spatial variability. The ASI instrument suite consists of sensors that measure ambient air temperature, pressure and probe acceleration, all of which have highly mature component options. Horizontal wind speed is another measurement that depends on a mature component, namely the Ultra-Stable Oscillator (USO), which is used to perform a Doppler Wind Experiment (DWE). The ASI and USO are expected to be also part of the primary probe and would enable comparison of wind shear at multiple locations.

The ASI includes an accelerometer used to measure the upper atmospheric structure during the atmospheric entry phase as the entry deceleration depends on the ambient density. The accelerometer is also used for inertial navigation to reconstruct the entry trajectory. Once the density vs altitude is known, assuming hydrostatic balance and ideal gas law will produce temperature and pressure as functions of altitude. Once the parachute is opened (typically at around the 100-mbar level), the entry aeroshell can be jettisoned so that the temperature and pressure sensors can be exposed to the environment and start taking their measurements. Capabilities to measure density and sound speed (Sect. 5.1) would increase the value of the ASI dataset, but these capabilities are not matured for outer planet exploration. USO ensures precise maintenance of the radio wave frequency transmitted by the probe to the orbiter so that any frequency change measured by the orbiter is dominated by the Doppler effect and not any instrumental artifacts. In a DWE, the orbiter must also carry an identical USO as a reference frequency source.

While measuring temperature, pressure and horizontal wind speeds at multiple locations using ASI and USO would be sufficiently valuable to justify secondary probes, a particularly high-priority measurement that currently lacks a mature suitable instrument option is variable concentrations of heavy-element molecules as functions of altitude. On prior atmospheric in-situ missions to Venus, Mars, Jupiter, and Titan, atmospheric composition measurements were carried out by a mass spectrometer, and Tunable Laser Spectrometers (TLS) have also been flown to Mars. However, a mass spectrometer tends to be a massive large instrument that tends to drive a probe design as illustrated in Sect. 6.1 and Table 3. TLS is also currently a large instrument. For example, the Sample Analysis at Mars (SAM) instrument suite on the Mars Science Laboratory (Mahaffy et al. 2012) combines a mass spectrometer and a TLS and weighs 40 kg (although this includes a Sample Manipulation System that would not be useful at Uranus). The objective to determine the spatial variability in their concentrations does not require all the capabilities of a large, heavy, mass spectrometer and TLS; in particular, a secondary probe does not need to measure the abundance of noble gases and isotopic ratios because they are expected to be spatially homogeneous (although xenon could be an exception if it condenses at Uranus, see Zahnle 2023). Thus, an instrument that exploits the chemical properties of the vapor molecules may offer the needed capability to measure the vapor concentrations. On the other hand, progress in miniaturizing TLS instrumentation (Webster et al. 2023) could enable a micro-TLS to perform compositional measurements aboard a miniaturized probe, since TLS data can be used to determine gas concentrations as well as isotope ratios.

Multiple efforts are ongoing to develop instruments that would enable vapor concentration measurements in Ice Giant atmospheres. Sensing mechanisms include functionalized field-effect transistors and chemiresistive sensors (Li et al. 2003; Sultana 2020; Ambrozik et al. 2020; Yaqoob and Younis 2021), microelectromechanical system (MEMS; Ba Hashwan et al. 2023), and quartz crystal microbalances (Alanazi et al. 2023). Some of these sensors have been space qualified and operated in space (Meyyappan 2015; Dawson et al. 2020); however, these technologies have not been demonstrated for conditions expected in giant planet atmospheres. Substantial development investment is needed to miniaturize sensors capable of satisfying the size and performance requirements for in situ exploration of Uranus. Further developments are also needed in designing inlet and sample processing system to ensure that the sensors are able to operate in the thermal conditions with potential presence of photochemical haze and condensed cloud droplets that may affect sensor operations (Wong 2017).

### Power, Heating, and Regulatory Requirements

Similar to larger probes, electrical power for secondary probe would be provided by onboard batteries. Due to the smaller overall mass of a secondary probe, the benefit of selecting a battery with higher energy density is relatively greater than for larger probes. Multiple battery technologies are available for future planetary science missions. Among them, lithium/ carbon monofluoride (Li/CF_*x*_) batteries may offer 640–700 Wh/kg energy density in a D-cell form factor (Surampudi et al. 2017; Krause et al. 2018), with a theoretical energy density of 2,596 Wh/kg (Bock et al. 2012). The typical lithium ion battery energy density is 145 Wh/kg. Table 3 lists different battery technologies assumed for different outer planets probe designs, and demonstrates that, for SNAP, incorporating Li/CF_*x*_ batteries allowed increasing the instrument mass fraction. The Europa Lander study also specified Li/CF_*x*_ batteries and called for development, since this technology does not have flight heritage (Hand et al. 2022).

Challenges in thermal management arise from the long dormant period each probe must withstand after being released from the orbiter, which is expected to last 30 days or longer. Without heating, the probe temperature would fall toward the radiative equilibrium temperature of ∼80 K around Uranus, which is much lower than the survival temperature of electronic components. Even though the heating power need is expected to be in the range of several watts (for SNAP, the estimated need is 3 W; Sayanagi et al. 2020), this represents a prohibitive amount of energy over a >30-day period. Thus, the only viable technology to satisfy this survival heating need is radioisotope heater units (RHUs), which utilize the radioactive decay heat release from plutonium-238 (NASA 2016). In principle, using RHUs in a mission incurs the regulatory nuclear launch safety fee (NASA 2022); however, any Flagship mission to Uranus is expected to incur the nuclear launch fee because it would carry a radioisotope thermoelectric generator (RTG) to provide electric power for the orbiter during the entire course of the mission. Incorporating RHUs in any secondary probe therefore will not represent additional cost in terms of nuclear launch safety fee, but schedule pressure must be considered (Zide et al. 2022) because payload nuclear components (including secondary probe RHUs) must be included in all design reviews required for nuclear launch safety approval.

## Conclusion

Multiple probe exploration of the giant planets is a concept that has enjoyed broad support from NASA and the science community since the Galileo Probe experiment was completed. As decadal surveys have grown more cost-conscious over the years, their explicit endorsement of multiprobes has waned, but the key science questions motivating in-situ exploration of Uranus continue to provide compelling justification for multiple probes.

Fletcher et al. (2020) provided justifications for targeting an atmospheric probe at Uranus into three locations: equatorial, mid-latitude, and polar domains. Given the desire to understand seasonal variation on Uranus, measurements in both north and south polar regions would be of immense value, justifying up to four atmospheric probe locations in total. Secondary probes would measure spatially-variable properties in these locations, complementing more detailed measurements in one of the locations conducted by a flagship-class probe with mass spectrometer and a more comprehensive instrument suite (Mandt et al. 2024). Although the focus of this specific paper is the science motivation for secondary probes at Uranus, we agree with the finding of Origins, Worlds, and Life 2022 that a mission with even a single probe would deliver uniquely powerful science return compared to an orbiter mission with only remote measurements.

Spatial variation in temperature has been observed in the stratosphere of Uranus (Rowe-Gurney et al. 2021), and multiple probes would be ideal for expanding our insight into how temperature may vary in the troposphere. In this deeper layer, heat transport by convection vs. radiation, measurable by atmospheric probes, could distinguish between very different evolutionary pathways and histories. Composition varies both spatially and temporally, and a more quantitative understanding would be enabled by multiprobe measurements capable of breaking degeneracies that affect remote sensing data from both spacecraft and observatories at the Earth. For example, spectroscopic retrievals of ammonia and methane concentrations are commonly affected by degeneracies with aerosol properties or with temperature variation. In situ measurements of composition and temperature can therefore provide anchor points for the modeling and interpretation of maps of spatial variation derived from remote sensing (Mandt et al. 2024). Although a single probe would effectively break degeneracies in remote sensing retrievals at the specific time and location of the probe entry, data from multiple probes would be a major advance. Multiprobe data would constrain physical models that could explain how dynamic processes differently affect distributions of temperature, composition, and aerosols throughout the atmosphere (Q7.2 and Q7.3 in Table 1). Understanding dynamic processes is ultimately necessary to constrain atmospheric abundances and thus planetary origins (Q7.1, Q1, Q2 in Table 1). We advocate that atmospheric structure measurements be expanded beyond only temperature and pressure to include density and sound speed, especially at Uranus where a means of quantifying the hydrogen ortho/para ratio would constrain both static stability and convective history.

There are no insurmountable barriers to multiprobe exploration of Uranus as part of the anticipated NASA flagship mission. The SNAP study (Sayanagi et al. 2020) demonstrated the feasibility a secondary probe on a flagship mission. The estimated $80M cost of a secondary probe is significant, but on the same order as a core facility instrument on the orbiter. A secondary probe could be included as a directed component of the mission, or the call for competed instruments could include a secondary probe within its scope. International collaboration—with one or several probes or probe components provided by another space agency—could be another pathway for achieving multiprobe exploration of Uranus.

Probe delivery to a separate location from the main flagship probe would require release from the orbiter on a separate orbit, which was shown to be feasible in the SNAP study. The situation would be further improved if aerocapture were included in the UOP mission design. Instrument maturity level for ASI and USO is high, although fully miniaturized versions of these components have not yet been flown on outer solar system atmospheric probes. The active development of miniaturized composition sensors, using chemiresistive or tunable laser spectroscopic approaches, must continue to be supported. Batteries with high energy density will enable a better science payload fraction. RHUs will be required for survival heating up to descent time, which argues for early integration of secondary probes into the overall mission design to ensure timely launch review and approval.

The first multiprobe mission to an outer planet atmosphere will represent a major increase in technical and scientific achievement in solar system exploration, compared to the single-probe Galileo exploration of Jupiter and Huygens exploration of Titan.

## Footnotes

1 Available as of 2024-Jan-04 on the NSPIRES website at https://nspires.nasaprs.com/external/solicitations/summary!init.do?solId={9D033998-EF04-4F71-9983-149581288481.
